# Coronary artery bypass grafting may not be suitable in pure myocardial bridging: a case report

**DOI:** 10.1093/ehjcr/ytag251

**Published:** 2026-04-09

**Authors:** Tsung-Lin Yang, Chuan-Chih Hsu

**Affiliations:** Department of Internal Medicine, School of Medicine, College of Medicine, Taipei Medical University, No. 250, Wuxing Street, Xinyi District, Taipei City 110, Taiwan; Division of Cardiology, Department of Internal Medicine, Taipei Medical University Hospital, No. 252, Wuxing Street, Xinyi District, Taipei City 110, Taiwan; Taipei Heart Institute, Taipei Medical University, No. 250, Wuxing Street, Xinyi District, Taipei City 110, Taiwan; Taipei Heart Institute, Taipei Medical University, No. 250, Wuxing Street, Xinyi District, Taipei City 110, Taiwan; Division of Cardiovascular Surgery, Department of Surgery, School of Medicine, College of Medicine, Taipei Medical University, No. 250, Wuxing Street, Xinyi District, Taipei City 110, Taiwan; Division of Cardiovascular Surgery, Department of Surgery, Taipei Medical University Hospital, No. 252, Wuxing Street, Xinyi District, Taipei City 110, Taiwan

**Keywords:** Myocardial bridging, Coronary artery bypass grafting, Angina, Case report

## Abstract

**Background:**

Myocardial bridging (MB) is a congenital coronary artery disease. Surgical interventions are indicated if angina persists after optimization of medical treatments. Coronary artery bypass grafting (CABG) is one of the surgical options for MB.

**Case summary:**

A 66-year-old male experienced exertional angina for 10 months. Treadmill test revealed ischaemic change during exercise. Coronary angiography revealed myocardial bridging in middle part of the left anterior descending artery (LAD) with prominent squeezing during systolic phase. Medical control was advised. However, symptoms persisted after maximally tolerated non-dihydropyridine calcium channel blockers and beta-adrenergic antagonists. After discussing with the heart team, the patient underwent CABG with left internal mammalian artery canalized to distal LAD. The surgeon chose CABG due to the concern of ventricular rupture after myotomy for long MB. Nevertheless, angina recurred and persisted several months after CABG. Follow-up coronary angiography delineated to-and-fro retrograde blood flow in graft without actual feeding flow into the distal LAD.

**Discussion:**

This case demonstrates that coronary artery bypass grafting may not be an appropriate therapeutic strategy for patients with isolated myocardial bridging. Comprehensive preoperative evaluation, including stress testing and invasive haemodynamic assessment, is crucial before considering CABG. Optimization of medical therapy for myocardial bridging–related symptoms should be thoroughly pursued prior to surgical intervention and should include beta-adrenergic blockers, non-dihydropyridine calcium channel blockers, ranolazine, and ivabradine. Nitrates should be avoided in patients with myocardial bridging. For patients with isolated myocardial bridging and refractory angina despite optimal medical therapy, surgical unroofing may represent a more favourable option than CABG.

Learning points

1. Stress testing and haemodynamic assessment are essential before proceeding with surgery in myocardial bridging patients on optimal medical treatment.
2. Unroofing is generally preferred over CABG in patients with isolated myocardial bridging without bystander coronary artery disease.

## Introduction

Myocardial bridging (MB) is a congenital coronary artery disease, in which situation coronary vessels are encased into myocardium, resulting in coronary vascular compression during myocardial contraction in systolic phase.^1^ The prevalence of MB is approximately 20% to 40% in the general population, and MBs are detected in around 10% of all coronary angiography examination.^2^ The medical treatment of choice is beta-adrenergic blocker (BB) or non-dihydropyridine calcium channel blocker (non-DHP CCB) among those who are intolerant to BB.^3^ For those who have refractory angina after maximally tolerated medication, revascularization including percutaneous coronary intervention, coronary artery bypass grafting (CABG) or myotomy (unroofing) can be another options.^4^

In this report, we introduce the clinical scenario and 1 year follow-up of a myocardial bridging patient with intractable angina who had undergone CABG.

## Summary figure

**Figure:**
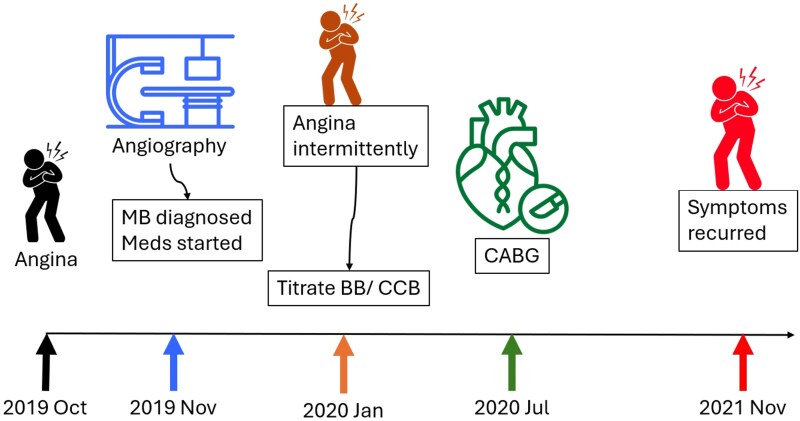


## Case presentation

**Figure 1:**
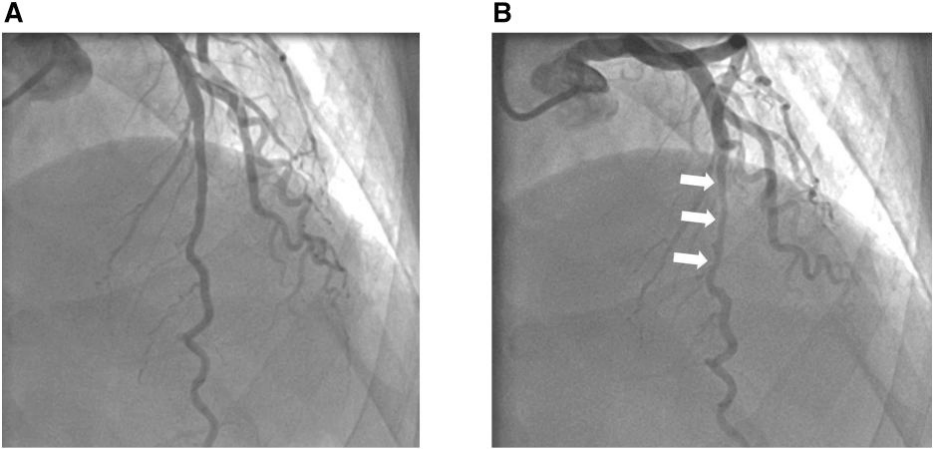
Title: diagnostic coronary angiography. *(A)* Left anterior descending artery in end-diastolic phase. *(B)* Myocardial bridging compressing the middle part of LAD (arrow) in end-systolic phase.

A 66-year-old male without remarkable underlying disease presented with intermittent chest tightness and exertional dyspnoea for 10 months. His symptoms were aggravated by physical activity and relieved with rest. A treadmill exercise test demonstrated significant ischaemic changes. Coronary angiography revealed a myocardial bridging, measuring approximately 23 mm in length at the mid-portion of the left anterior descending (LAD) artery, without significant atherosclerotic lesions (*Figure 1A*: End-diastolic phase, and 1B: end-systolic phase).

Medical therapy with a beta-adrenergic blocker and a non-dihydropyridine calcium channel blocker was initiated, while nitrates were avoided. Despite administration of maximally tolerated doses, chest discomfort recurred repeatedly. Over the subsequent nine months, his angina relapsed unpredictably and remained poorly controlled. The medical team was sure that the persistent symptoms were not nitrate-related, as nitrates had been avoided in the management of myocardial bridging. After discussion within the heart team, including cardiologists and cardiovascular surgeons, the patient elected to undergo coronary artery bypass grafting (CABG) for refractory angina attributed to myocardial bridging.

The cardiovascular surgeon chose CABG over unroofing because the patient’s myocardial bridging segment was relatively long, making unroofing potentially associated with a risk of ventricular rupture. The surgery was performed via standard longitudinal sternotomy plus pericardiotomy, with the left internal mammary artery (LIMA) anastomosed to the left anterior descending artery distal to the bridging segment in an end-to-side fashion. The aortic cross-clamp time was 68 min, and the cardiopulmonary bypass time was 85 min.

Postoperatively, the patient experienced marked improvement in chest pain and became asymptomatic immediately after surgery. He was discharged one week after CABG. Discharge medications included aspirin 100 mg/day, bisoprolol 1.25 mg/day, verapamil 240 mg twice daily, rosuvastatin 10 mg/day, and metformin 500 mg/day.

**Figure 2:**
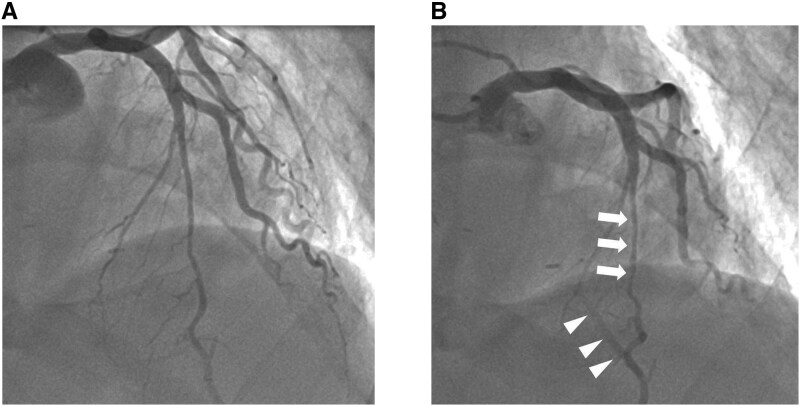
Title: follow-up coronary angiography after bypass grafting. *(A)* Left anterior descending artery in end-diastolic phase. *(B)* Myocardial bridging compressing the middle part of LAD (arrow) in end-systolic phase, which resulted in reverse blood flow into left internal mammary artery grafting (arrowhead).

The patient was followed regularly in the outpatient clinic and remained clinically stable initially. However, approximately 1 year after surgery, he began to experience recurrent chest compression during strenuous daily activities, such as lifting heavy objects or climbing stairs. Anginal symptoms persisted despite further optimization of medical therapy. Repeat coronary angiography was therefore performed (*Figure 2A*: end-diastolic phase, and 2B: end-systolic phase).

Angiography demonstrated persistent vascular compression caused by the myocardial bridge. Prominent systolic flow reversal was observed both in the segment proximal to the bridged LAD and within the LIMA graft, resulting in a to-and-fro flow pattern in the graft. As the graft remained patent without evidence of stenosis or obstruction, the patient elected to continue conservative medical management without further intervention.

## Discussion

Currently, there are no strong surgical guidelines to determine whether CABG or unroofing should be selected for patients with myocardial bridging. A study has suggested that CABG may be more suitable for patients with long or deep myocardial bridges, which was also an important factor considered by our medical team when making the treatment decision.^5^ However, this case illustrates the limited therapeutic efficacy of CABG in patients with isolated myocardial bridging. Despite angiographic confirmation of a patent graft and native coronary arteries, the patient continued to experience angina. The fundamental mechanism underlying the persistent symptoms was inadequate restoration of effective blood flow to the distal LAD. Follow-up angiography revealed significant competitive flow between the native LAD and the LIMA graft, resulting in inefficient myocardial perfusion.

Previous studies have reported a 1-year internal mammary artery graft failure rate of 30–50% in the presence of competitive flow.^6–8^ In general, the more severe the native coronary artery stenosis at the time of internal mammary artery grafting, the lower the likelihood of subsequent graft occlusion.^8,9^ Conversely, higher native coronary flow pressure increases the risk of graft failure due to competitive flow. In the present case, robust systolic compression from the myocardial bridge forced retrograde flow within the LIMA graft, creating a competitive circulation pattern. To a certain extent, blood stagnated within the internal mammary artery rather than progressing forward.^8,9^

Marked systolic flow reversal was observed both proximal to the myocardial bridge and within the graft, resulting in haemodynamic conflict between the left internal mammary artery and the left subclavian artery. Consequently, not only was distal coronary perfusion compromised but myocardial efficiency may also have been impaired by internal haemodynamic friction and wasted contractile effort. Although CABG is considered a surgical option for medication-refractory angina in myocardial bridging, chest pain in this patient recurred and progressively worsened over time despite surgical revascularization.

The apparent perioperative anti-anginal effect of CABG in this case may, at least in part, be attributable to postoperative analgesia, alterations in coronary flow dynamics, transient reduction in myocardial workload, and temporary heart rate control, rather than to sustained haemodynamic improvement.

In contrast to coronary bypass grafting, surgical myotomy (muscular unroofing) may represent a more appropriate intervention for selected patients with myocardial bridging. Direct comparative evidence between CABG and unroofing remains limited. To date, no prospective randomized trials or guideline recommendations specifically address the optimal surgical management of isolated myocardial bridging. Retrospective studies have suggested that unroofing may be associated with superior clinical outcomes compared with CABG in patients with hypertrophic cardiomyopathy and concomitant myocardial bridging.^10^ Another retrospective analysis reported favourable mid-term outcomes with unroofing for symptomatic LAD myocardial bridging and suggested reserving CABG for cases with concomitant proximal coronary artery stenosis.^11^ More evidence is needed, including pharmacologic strategies and surgical approaches, to guide the optimal management of relieving clinical symptoms, improving quality of lives, and reducing major adverse cardiac events among MB patients. This valuable clinical experience of the current case has shown us CABG may not be suitable in pure myocardial bridging.

This case highlights several important pitfalls. First, functional stress imaging studies—including stress echocardiography, myocardial perfusion scintigraphy, or stress cardiac magnetic resonance imaging—were not performed. Second, invasive physiological assessments such as instantaneous wave-free ratio (iFR) or fractional flow reserve (FFR) were not obtained during coronary angiography. Recent evidence suggests that adenosine-induced diastolic FFR may reliably identify the haemodynamic significance of myocardial bridging.^12^ Angiography alone may therefore be insufficient for surgical decision-making. Third, newer anti-anginal agents, particularly ranolazine and ivabradine, which are recommended in recent European Society of Cardiology guidelines, were not trialled.^13^ Without comprehensive medical optimization, the indication for CABG in this patient remained incomplete.

## Conclusion

In this case, we describe a patient with isolated myocardial bridging and refractory angina despite maximally tolerated beta-adrenergic blockers and non-dihydropyridine calcium channel blockers. Although coronary artery bypass grafting initially provided symptomatic relief, angina subsequently recurred. Follow-up coronary angiography demonstrated competitive flow between the left internal mammary artery graft and the myocardial bridge, a haemodynamic pattern that predisposes to graft dysfunction and persistent ischaemic symptoms. These findings suggest that CABG may not be an appropriate treatment strategy for isolated myocardial bridging. In contrast, surgical unroofing may represent a more effective option for selected patients with myocardial bridging.

## Supplementary Material

ytag251_Supplementary_Data

## Data Availability

The data videos underlying this article are available in the article and in its online Supplementary material.

## Lead author biography

Hi! I am Dr. Tsung-Lin Yang from Taiwan. I am a cardiolgist, specialized in transcatheter coronary intervention and echocardiography, including trans-oesophageal echocardiography. My study of interest is myocardial bridging, which has many interesting properties that need further researches. Interested in myocardial bridging? Please contact me and let's study together ∼

## Notes

**Consent:** The authors confirm that written consent for submission and publication of this case report including images and associated text has been obtained the patient, in line with COPE guidance.

This patient had signed informed consent for publication presentation. This report was approved by the Institutional Review Board of the Taipei Medical University Hospital (N202403028).
